# Stigmatisation des personnes vivant avec le VIH dans les services de soins au Togo

**DOI:** 10.48327/mtsi.v3i2.2023.260

**Published:** 2023-04-04

**Authors:** Julienne Noude TÉCLESSOU, Abla Séfako AKAKPO, Augustin Kokouvi DOKLA, Damien Kégnidé AMOUSSOU, Kodzo DEKU, Charles Abalo LIMAIE, Vincent Palokinam PITCHÉ

**Affiliations:** 1 Faculté des sciences de la santé, Université de Lomé, 08 B.P. 8615 Lomé, Togo; 2 Réseau national des associations de personnes vivant avec le VIH (RAS+ Togo), Togo; 3 Conseil national de lutte contre le sida et les IST du Togo (CNLS-IST), Lomé, Togo

**Keywords:** Stigmatisation, PvVIH, Services de soins, Togo, Afrique subsaharienne, Stigma, PLHIV, Health care services, Togo, Sub-Saharan Africa

## Abstract

**Introduction:**

L’élimination du sida en tant que menace pour la santé publique implique une prise en charge globale des personnes dépistées dans toutes les structures sanitaires et en dehors de toute discrimination/stigmatisation. Le but de cette étude était d’évaluer les problèmes de stigmatisation et de discrimination des personnes vivant avec le VIH (PvVIH) dans les structures de soins et leurs implications dans l'accès aux soins de ces PvVIH.

**Méthodologie:**

Il s'agit d'une étude transversale menée dans les 6 régions sanitaires du Togo ciblant les personnes âgées de 18 ans et plus, vivant avec le VIH. Un échantillonnage hybride associant un référencement en chaîne limité et un échantillonnage basé sur la cartographie des lieux de rencontre/d'activités a été utilisé. Les données ont été collectées à l'aide du questionnaire digitalisé de la version 2.0 de l'index de stigmatisation des PvVIH.

**Résultats:**

Au total, 1 119 PvVIH d’âge moyen de 39 ans ont été incluses. Le sexe-ratio était de 0, 5, et 43, 4% connaissaient leur séropositivité depuis 1 à 4 ans. Au cours des 12 derniers mois les expériences de stigmatisation rapportées par les PvVIH de la part du personnel de soins étaient: les commérages (13%), la divulgation du statut sans le consentement (10%), ou le refus de tout contact physique (2, 6%). En matière de santé reproductive, il avait été conseillé à 2, 1% des enquêtés de ne pas devenir père/mère et 1, 4% avaient vu leur accès aux antirétroviraux (ARV) conditionné par l'utilisation d'une méthode contraceptive. Par ailleurs, parmi ceux qui étaient sous ARV, 28, 4% avaient manqué une fois leur dose de traitement au cours des 12 derniers mois par crainte qu'une personne apprenne leur séropositivité. Enfin, 39, 5% des enquêtés qui n’étaient pas sous ARV avaient la crainte que les travailleurs de santé les traitent mal ou révèlent leur séropositivité sans leur consentement.

**Conclusion:**

En milieu de soins, ce phénomène de stigmatisation lié au VIH est protéiforme et mérite d’être documenté afin d’être pris en compte pour améliorer la qualité des offres de services aux populations bénéficiaires.

## Abstract (en)

**Introduction:**

The elimination of AIDS as a threat to public health implies comprehensive care for those tested in all health facilities and without discrimination/stigmatization. The aim of this study is to evaluate the problems of stigmatization and discrimination of people living with HIV (PLHIV) in health care facilities and their implications for access to care for PLHIV.

**Methodology:**

This is a descriptive cross-sectional study in the 6 health regions of Togo targeting people aged 18 years and over, living with HIV. A hybrid sampling method combining a limited chain referral and a sampling based on the mapping of meeting places/activities was used. Data were collected using the digitized version 2.0 of the PLHIV Stigma Index questionnaire.

**Results:**

A total of 1 119 PLHIV with a mean age of 39 years were included. The sex-ratio was 0.5, and 43.4% had known their HIV status for 1 to 4 years. In the last 12 months, the experiences of stigmatization reported by the PLHIV from health personnel were: gossip (13%), disclosure of status without consent (10%) or avoidance of physical contact (2.6%). In terms of reproductive health, 2.1% of respondents had been advised not to become fathers/mothers and 1.4% had their access to antiretroviral drugs (ARV) conditioned by the use of a contraceptive method. 28.4% of those on ARV had missed one dose of treatment in the last 12 months for fear that someone would find out they were HIV positive. Also, 39.5% of the respondents who were not on ARV justified this by the fear that health workers would treat them badly or reveal their HIV status without their consent.

**Conclusion:**

In health care settings, this phenomenon of HIV-related stigma is multifaceted and deserves to be documented so that it can be taken into account in order to improve the quality of services offered to beneficiary populations.

## Introduction

L’élimination du sida en tant que menace pour la santé publique implique une prise en charge globale des personnes dépistées tant pour leurs problèmes de santé liés au VIH que pour tout autre problème de santé n'ayant pas un lien direct avec leur séropositivité. Cette prise en charge doit se faire dans toutes les structures sanitaires et en dehors de toute discrimination/stigmatisation. La stigmatisation est un processus d’étiquetage, de stéréotypage, de séparation, de perte de statut et de discrimination d'individus ou de groupes [8]. Deux formes de stigmatisation sont souvent décrites: la stigmatisation par une tierce personne et l'auto-stigmatisation. La stigmatisation par une tierce personne peut survenir tant dans l'environnement socio-professionnel que familial, mais aussi sanitaire où elle est exercée par un prestataire de soins. Plusieurs études ont rapporté que la stigmatisation et la discrimination liées au VIH envers les personnes vivant avec le VIH (PvVIH) se produisent au sein des familles, des communautés et des établissements de santé [6, 7, 13, 20, 22]. Ces problèmes de stigmatisation et de discrimination constituent un goulot d’étranglement dans la mise en œuvre des interventions sur le VIH et la santé en général; c'est ce qui a amené l'ONUSIDA à en faire un des objectifs dans le cadre du Plan mondial de lutte contre le sida depuis une dizaine d'années [23].

Des actes de stigmatisation et de discrimination à l’égard des PvVIH ont été signalés dans des établissements de soins de diverses manières: refus de soins, commérage, négligence, référence inutile vers d'autres établissements de soins [1, 2, 12, 13, 18]. La stigmatisation interne ou l'auto-stigmatisation est l'acceptation de la perception de son statut inférieur au sein de la société et se manifeste par une faible estime de soi, l'isolement et le retrait social [9, 11].

Le but de cette étude était d’évaluer la stigmatisation et la discrimination des PvVIH dans les structures de santé et leurs implications dans l'accès aux soins de ces PvVIH au Togo.

## Méthodologie

### Type et cadre d’étude

Il s'est agi d'une étude transversale descriptive et analytique nationale qui s'est déroulée dans l'ensemble des 44 districts des 6 régions sanitaires du Togo. L'enquête s'est déroulée sur les sites de prise en charge des PvVIH. La sélection des sites d'enquête était basée sur l'approche « localisation et population de l'ONUSIDA ». Étaient donc pris en compte: la localisation urbaine ou rurale du site, le poids de la file active des PvVIH de chaque région sanitaire dans la file active nationale (fardeau de l’épidémie) et l'existence d'organisations communautaires pour les PvVIH dans les régions/districts sanitaires.

### Population de l’étude

La population d’étude était constituée par les PvVIH âgées de 18 ans et plus connaissant leur statut sérologique depuis plus de 12 mois. Il s'agissait des PvVIH suivies ou non dans des structures de prise en charge médicale.

### Échantillonnage

Un échantillonnage hybride a été utilisé associant un échantillonnage déterminé par les répondants *(Respondent-Driven Sampling/RDS* ou boule de neige non probabiliste), un échantillonnage basé sur la cartographie des lieux de rencontre/d'activités (*Time Location Sampling/TLS*), le choix aléatoire/systématique et le choix raisonné/commodité/convenance.

En nous basant sur les données de l'enquête 2014 de l'Index de stigmatisation, nous émettons l'hypothèse qu'environ 1 PvVIH sur 16 (p = 6, 3%) pourrait décider d’éviter de rechercher des soins de santé en raison de la stigmatisation anticipée basée sur le statut VIH. La formule de Schwartz a été utilisée pour l'estimation de la taille de l’échantillon:

- n: taille d’échantillon minimale requise exprimée en nombre d'individus de la population cible;
- p: valeur de référence de l'indicateur visé, fixée à 6, 3% pour rester dans une optique conservatrice de maximisation de la taille de l’échantillon;
- δ: marge d'erreur fixée pour l'ensemble des indicateurs à 3%;
- α: niveau de signification statistique fixée pour l'ensemble des indicateurs à 95%;
- *deff*: effet de grappe fixé à 2.
  La taille minimale de l’échantillon est donc de 1 008 PvVIH. En assumant 10% de non-réponses (soit 102 PvVIH additionnelles), une taille ajustée de 1 110 a été considérée.

### Collecte des données

Le questionnaire utilisé est celui élaboré par le partenariat international pour l'Index de la stigmatisation des personnes vivant avec le VIH (Stigma Index 2.0) [14]. Une phase préparatoire a permis d'informer les acteurs sur le terrain, de former des enquêteurs et de réaliser un prétest.

### Analyse des données

L'analyse des données a été effectuée à l'aide du logiciel SPSS V23.0. Les données ont systématiquement été désagrégées en fonction des caractéristiques générales des enquêtés et d'autres variables d'intérêt en fonction du contexte. Une analyse comparative des proportions observées a été réalisée en utilisant le test de Chi 2 pour échantillons indépendants. Le seuil de validation des tests a été fixé à 95%.

### Consentement et aspects éthiques

Le protocole de l’étude a été approuvé par le Comité bioéthique pour la recherche en santé du Ministère de la santé du Togo (avis N° 015/CBRS du 25 septembre 2020). Toutes les données ont été collectées et traitées de façon anonyme. Une note d'information et un formulaire de consentement éclairé ont été signés par chaque enquêté.

## Résultats

Au total, 98 sites répartis sur l'ensemble des 6 régions sanitaires ont été sélectionnés. Sur ces sites, 1 119 PvVIH issues de la population générale et des sous-types ont été incluses.

L’âge médian des enquêtés était de 39 ans avec des extrêmes de 18 ans et 80 ans. La tranche d’âge de 30-39 ans était la plus représentée (28, 2%), suivie des 40 à 49 ans (26, 5%). Selon le sexe biologique, 747 (66, 8%) enquêtés étaient des femmes soit un sexe-ratio de 0, 5. Selon l'identité de genre, les enquêtés s'identifiant comme « femmes » représentaient 777 (69, 4%), les transgenres 36 (3, 2%), et les non-binaires 5 (0, 4%). Quatre cent quatre-vingt-dix (43, 4%) enquêtés connaissaient leur séropositivité depuis 1 à 4 ans.

### Expériences de stigmatisation de la part du personnel de santé

Parmi les 1 119 enquêtés, 461 s’étaient rendus au moins une fois dans les structures de soins (publiques ou privées) au cours des 12 derniers mois. Plusieurs formes de stigmatisation ont été rapportées par ces 461 enquêtés à cause de la séropositivité. Il s'agissait: des commérages (13%), des violences verbales (12, 1%), de la divulgation du statut sans le consentement (10%) (Tableau I). Douze des 461 enquêtés (2, 6%) avaient rapporté que les personnels de santé évitaient tout contact physique avec eux ou prenaient des précautions supplémentaires (port de gants en double), et 3, 9% s’étaient vu refuser des soins de santé en raison de leur séropositivité. Le refus de services de santé, les violences verbales et les violences physiques à cause de la séropositivité étaient plus souvent observées chez les femmes (respectivement 55, 6% (p = 0, 04), 76, 8% (p = 0, 001) et 71, 4% (p = 0, 01)). Par ailleurs, 121 des 1 119 enquêtés (10, 8%) affirmaient ne pas savoir si leur dossier médical était tenu confidentiel et 10 (0, 9%) enquêtés étaient certains que leur dossier médical ne l’était pas resté.

**Tableau I:** Stigmatisation des enquêtés par les agents de santé Stigmatization of respondents by health care givers

Items	N (patients victimes)	%
**Stigmatisation des PvVIH de la part du personnel de santé de leur structure de prise en charge au cours des 12 derniers mois (n = 461)**		
Commérages à cause de sa séropositivité	60	13
Violence verbale à cause de sa séropositivité	56	12,1
Divulgation de sa séropositivité à d'autres personnes sans son consentement	46	10
Conseil de ne pas avoir de relations sexuelles à cause de sa séropositivité	27	5,9
Refus de services de santé en raison de sa séropositivité	18	3,9
Victime de violence physique à cause de sa séropositivité	14	3
Évitement de tout contact physique/prise de précautions supplémentaires (port de gants en double)	12	2,6
**Opinion des enquêtés sur la tenue confidentielle du dossier médical lié à leur statut sérologique (n = 1 119)**		
Je suis certain(e) que mon dossier médical est tenu confidentiel et qu'il ne sera pas partagé sans mon consentement éclairé et écrit	988	88,3
Je ne sais pas si mon dossier médical est tenu confidentiel	121	10,8
Je suis certain que mon dossier médical n'est pas tenu confidentiel	10	0,9
**Stigmatisation en matière de santé sexuelle et reproductive de la part des agents de santé au cours des 12 derniers mois (n = 1 119)**		
A été conseillé de ne pas devenir mère/père d'un enfant	24	2,1
S'est vu conditionner l'accès aux traitements ARV par l'utilisation de la contraception ou d'une méthode contraceptive en particulier	16	1,4
A été victime d'un refus de la contraception ou des services de planification familiale	12	1,1
A été stérilisé à son insu ou sans son consentement	5	0,4
A subi une pression ou a été incité à se faire stériliser	3	0,3
**Enquêtés de sexe féminin rapportant une expérience de stigmatisation en matière de santé sexuelle et reproductive de la part d'un professionnel de santé (n = 737)**		
A subi une pression pour le suivi d'un traitement antirétroviral pendant la grossesse afin de réduire les chances de transmission du VIH	80	10,9
A subi une pression pour l'utilisation d'une pratique d'alimentation du nourrisson en particulier	34	4,6
A subi une pression pour l'utilisation d'une méthode contraceptive en particulier	20	2,7
A subi une pression pour l'utilisation d'une méthode/option d'accouchement en particulier	18	2,5
A été conseillée d'interrompre une grossesse	16	2,2
**Violation de droits liée à l'obligation de passer un dépistage du VIH ou de dévoiler leur statut pour bénéficier d'un service essentiel (n = 1 119)**		
A été obligé(e) de passer un dépistage du VIH ou de dévoiler son statut pour recevoir des soins de santé	85	7,6
A été obligé(e) de passer un dépistage du VIH ou de dévoiler son statut pour obtenir un visa ou déposer une demande de résidence/nationalité dans un pays	50	4,5
A été obligé(e) de passer un dépistage du VIH ou de dévoiler son statut pour postuler pour un emploi ou souscrire un régime de pensions	46	4,1
A été obligé(e) de passer un dépistage du VIH ou de dévoiler son statut pour fréquenter un établissement scolaire ou obtenir une bourse	34	3
A été obligé(e) de passer un dépistage du VIH ou de dévoiler son statut pour obtenir une assurance maladie	30	2,7
**Connaissance de l'existence des lois protégeant les PvVIH contre la discrimination dans le pays**		
Oui il existe des lois	480	42,9
Je ne sais pas s'il existe des lois	639	57,1
**Actions entreprises pour le changement en matière de lutte contre la stigmatisation et/ou discrimination (n = 1 119)**		
A interpellé ou éduqué quelqu'un qui faisait de la stigmatisation et/ou de la discrimination contre d'autres personnes vivant avec le VIH	402	35,9
A fourni un soutien émotionnel, financier ou autre à une personne vivant avec le VIH pour l'aider à gérer la stigmatisation et/ou la discrimination	307	27,4
A interpellé ou éduqué quelqu'un qui faisait de la stigmatisation et/ou de la discrimination contre lui	291	26
A rejoint une association ou participé à une campagne d’éducation pour répondre à la stigmatisation et à la discrimination à l'endroit des personnes vivant avec le VIH	181	16,2
A encouragé un responsable communautaire à passer à l'action concernant des enjeux de stigmatisation et de discrimination qui touchent les personnes vivant avec le VIH	179	16
A parlé aux médias d'enjeux de stigmatisation et de discrimination qui touchent des personnes vivant avec le VIH	75	6,7
A encouragé un responsable gouvernemental ou politique à passer à l'action concernant des enjeux de stigmatisation et de discrimination qui touchent les personnes vivant avec le VIH	74	6,6

### Stigmatisation en matière de santé sexuelle et reproductive de la part des agents de santé

Concernant la santé sexuelle, il avait été conseillé à 24 des 1 119 enquêtés (2,1%) de ne pas devenir mère/père d'un enfant et 16 (1,4%) avaient vu leur accès aux traitements antirétroviraux (ARV) conditionné par l'utilisation d'une méthode contraceptive. Parmi les 737 enquêtées de sexe féminin, il avait été conseillé, au moins une fois, à 16 d'entre elles (2,2%) d'interrompre une grossesse dont 0,8% au cours des 12 derniers mois précédant l'enquête. De plus,80 des 737 (10,9%) avaient subi une pression pour le suivi d'un traitement ARV pendant la grossesse afin de réduire les probabilités de transmission du VIH dont 4,9% au cours des 12 derniers mois (Tableau I). Quatre-vingt-cinq (7,6%) des 1 119 enquêtés dont 3,6% dans les 12 derniers mois, ont été obligés de dévoiler leur statut pour recevoir des soins de santé.

### Stigmatisation intériorisée et résilience des enquêtés

Au cours des 12 derniers mois précédant l'enquête, les comportements d'auto-stigmatisation étaient: décider de ne pas avoir de relations sexuelles (405 enquêtés soit 36,2%); éviter d'aller dans un centre de santé en cas de besoin (204 enquêtés soit 18,2%); ou encore choisir de ne pas avoir recours à un soutien social (211 soit 18,9%). Trois cent onze (27,8%) s'isolaient de la famille et/ou amis,321 (28,7%) ne participaient pas à des rencontres sociales et 135 (12,1%) ne postulaient pas à un emploi.

### Interactions avec les services de soins de santé

#### Dépistage et mise sous antirétroviraux (ARV)

Huit cents enquêtés (71,5%) avaient confirmé le caractère volontaire de leur dépistage: « c’était leur choix », et 138 (12,3%) affirmaient avoir été dépistés à leur insu et ne l'avoir su qu'après (Tableau II). Les raisons principales pour lesquelles les enquêtés ont été soumis au dépistage du VIH étaient qu'un prestataire le leur a recommandé, le leur a effectué dans le cadre d'autres soins de santé (325 soit 29%), ou le fait de se sentir malade et d'avoir pensé que c’était le VIH (307 soit 27,4%). Plus de la moitié (532 soit 66,5%) des enquêtés qui avaient fait le choix du dépistage du VIH avaient hésité par peur des réactions des autres suite à un éventuel résultat positif.

**Tableau II:** Interaction avec les services de santé Interaction with health services

Items	N (patients victimes)	%
**Caractère volontaire du dépistage du VIH (n = 1 119)**		
Oui c’était mon choix	**800**	**71,5**
Oui c’était sous pression d'autres personnes	**151**	**13,5**
Non j'ai été dépisté(e) à mon insu et je ne l'ai su qu'après	**138**	**12,3**
Non j'ai été forcé(e) au dépistage sans mon consentement	**19**	**1,7**
Non je suis né(e) avec le VIH ou je l'ai contracté dans la petite enfance, je ne savais pas qu'on m'avait dépisté(e)	**11**	**1**
**Principale raison de réalisation du test de dépistage du VIH (n = 1 119)**		
Un prestataire me l'a recommandé, ou on me l'a effectué dans le cadre d'autres soins de santé	**325**	**29**
Je me sentais malade et j'ai pensé (ou ma famille a pensé) que c’était peut-être lié au VIH	**307**	**27,4**
Je croyais être une personne à risque	**157**	**14**
Dans le cadre d'un programme communautaire	**95**	**8,5**
C’était une exigence	**31**	**2,8**
Je voulais savoir tout simplement	**115**	**10,3**
Autres raisons	**89**	**7,9**
**Temps écoulé entre le désir et la réalisation du premier test de dépistage du VIH (n = 1 119)**		
Inférieur à 6 mois	**667**	**59,6**
6 mois à 2 ans	**143**	**12,8**
Plus de 2 ans	**157**	**14**
Ne sait pas/Ne se souvient pas	**152**	**13,6**
**Raisons d'hésitation, de retard ou d'empêchement à entreprendre un traitement ARV (n = 43)**		
Pas prêt à faire face à mon statut	**29**	**67,4**
Crainte que mon (ma) partenaire, famille ou amis découvrent mon statut	**27**	**62,8**
Crainte que d'autres personnes (outre famille/amis) découvrent mon statut	**27**	**62,8**
Crainte que les personnels de santé me traitent mal ou révèlent ma séropositivité sans mon consentement	**17**	**39,5**
Ai eu une mauvaise expérience avec un agent de santé auparavant	**16**	**37,2**
**Consentement des enquêtés à l'initiation d'un traitement antirétroviral (n = 1 076)**		
On m'a parlé des bienfaits et j'ai choisi de commencer dès qu'il m'a été proposé	**928**	**86,2**
Lorsque le traitement m'a été proposé, j'ai décidé d'attendre et je l'ai commencé plus tard	**97**	**9**
Des agents de santé ont fait pression sur moi ou m'ont forcé(e) à commencer le traitement	**27**	**2,5**
Autres raisons	**24**	**2,2**
**Temps écoulé entre le diagnostic de VIH et le début du traitement antirétroviral (n = 1 076)**		
Traitement débuté immédiatement ou le jour même du diagnostic	**277**	**25,7**
1 jour à 1 mois	**450**	**41,8**
1 à 6 mois	**190**	**17,7**
6 mois à 2 ans	**61**	**5,7**
Plus de 2 ans	**62**	**5,8**
Je ne me souviens pas	**37**	**3,4**
**Interruption du traitement ARV au cours des 12 derniers mois pour des raisons liées à la stigmatisation (n = 276)**		
Peur que quelqu'un découvre mon statut VIH	**66**	**23,9**
Pas prêt à faire face à mon infection à VIH	**57**	**20,6**
N'ai pas suivi ou ai cessé de suivre un traitement contre le VIH au cours des 12 derniers mois	**21**	**7,6**
Crainte que les professionnels de santé me traitent mal ou divulguent mon statut VIH sans mon consentement	**10**	**3,6**
Me suis vu refuser un traitement contre le VIH en raison de ma consommation actuelle de drogue	**1**	**0,4**
Autres raisons	**140**	**50,7**
**Raisons d'hésitation, de retard ou d'empêchement de reprendre les soins ou le traitement contre le VIH (n = 276)**		
Pas prêt à faire face à mon infection au VIH	**113**	**40,9**
Crainte que d'autres personnes (outre ma famille ou mes amis) découvrent mon statut	**107**	**38,8**
Crainte que mon (ma) partenaire, famille ou amis découvrent mon statut	**91**	**33**
Crainte que les agents de santé me traitent mal ou révèlent ma séropositivité sans mon consentement	**49**	**17,8**
Ai eu une mauvaise expérience avec un agent de santé auparavant	**44**	**15,9**
**Lieu habituel de recours aux soins et traitements pour le VIH (n = 1 076)**		
Clinique/hôpital public	**452**	**42**
Clinique/hôpital privé ou docteur	**94**	**8,7**
Clinique ou établissement non gouvernemental	**505**	**46,9**
Soins dirigés par la communauté	**9**	**0,8**
Endroits multiples	**3**	**0,3**
**Connaissance d'une clinique accessible offrant des services liés au VIH, dirigée par la communauté (n = 1 119)**		
Oui il y en a une mais je n'y ai pas accès pour mes soins	**350**	**31,3**
Oui il y en a une et j'y ai accès pour mes soins VIH	**308**	**27,5**
Non il n'y en a pas	**397**	**5,7**
Je ne sais pas	**64**	**35,5**
**Services liés au VIH accessibles dans le centre communautaire (n = 658)**		
Information sur le VIH	**582**	**88,4**
Soutien par les pairs	**459**	**69,8**
Conseil en matière d'adhésion	**40**	**6,1**
Service et produits de prévention	**461**	**70,1**
Service de traitement du VIH	**590**	**89,7**
Gestion des dossiers	**441**	**67**
Services de soins et de dépistage du VIH	**466**	**70,8**

La majorité (1 076 soit 96,2%) des enquêtés étaient sous traitement ARV au moment de l'enquête. Les principales raisons avancées par les 43 enquêtés qui n’étaient pas sous ARV étaient: « ne pas être prêt à faire face à leur infection au VIH » (29 soit 67,4%); « la crainte que d'autres personnes découvrent leur statut » (27 soit 62,8%), ou « la crainte que les travailleurs de santé les traitent mal ou révèlent leur séropositivité sans leur consentement » (17 soit 39,5%) (Tableau II).

Parmi les enquêtés ayant consenti à l'initiation des ARV,928 (86,2%) avaient entendu parler des bienfaits du traitement et choisi de commencer dès qu'il le leur a été proposé. Par contre, pour 27 (2,5%) enquêtés, des agents de santé avaient fait pression sur eux et les avaient forcés à commencer le traitement.

#### Interruptions de traitement

Parmi les 1 076 enquêtés sous ARV,276 (25,6%) les avaient déjà interrompus au moins une fois dont principalement les femmes (69,2%). Les enquêtés ayant arrêté les soins ou le traitement contre le VIH avançaient comme raisons de la non-reprise des soins, le fait de ne pas être prêts à faire face à leur infection au VIH (40,9%), ou encore la crainte que les agents de santé les traitent mal ou révèlent leur séropositivité sans leur consentement (17,8%).

Le recours aux soins et traitements ARV par les 1 076 enquêtés se faisait essentiellement dans les structures associatives et communautaires (46,9% des enquêtés), dans les structures de soins publiques (42% des enquêtés), mais aussi dans les cliniques privées (11,1%). 31,3% des enquêtés déclaraient connaître des structures associatives et communautaires accessibles offrant des services liés au VIH, dirigées par la communauté, mais n'y ont pas accès pour des soins (Tableau II).

### Droits de la personne et actions pour le changement

Quatre cent quatre-vingts (42,9%) des 1 119 enquêtés qui n'avaient subi aucune violation de leurs droits au cours des 12 derniers mois avaient connaissance de l'existence des lois protégeant les PvVIH contre la discrimination dans le pays. En outre,402 (35,9%) avaient rapporté avoir interpellé ou sensibilisé quelqu'un qui faisait de la stigmatisation et/ou de la discrimination contre d'autres personnes vivant avec le VIH.

## Discussion

Les problèmes de stigmatisation et de discrimination constituent l'un des enjeux importants dans l'accélération de la réponse au VIH dans les pays d'Afrique de l'Ouest et centrale. Si ces problèmes restent prépondérants dans les milieux familial et professionnel [6, 7, 13, 20, 22], les résultats de notre enquête montrent que ce phénomène est non négligeable dans les structures de soins avec des impacts négatifs importants pour les PvVIH en matière d'accès aux soins et pour l'efficacité de la lutte contre le VIH dans le pays. En milieu de soins, ce phénomène de stigmatisation lié au VIH est protéiforme et mérite d’être documenté afin d’être pris en compte pour améliorer la qualité des offres de services aux populations bénéficiaires.

Quarante-six (10%) des 461 enquêtés qui s’étaient rendus dans les structures de soins avaient vu leur statut sérologique divulgué par les agents de santé sans leur consentement. Bien que nous n'ayons pas recherché les raisons de cette divulgation de statut dans notre étude, plusieurs raisons peuvent expliquer la divulgation de sérologie sans le consentement des patients. Ainsi, en 1996 en Afrique du Sud, G. Seidel *et al.* avaient évoqué la divulgation du statut par les personnels de santé se justifiant par la difficulté à trouver l’équilibre entre le secret médical des PvVIH et la nécessité d'aider et de protéger les personnes de leur entourage [24].

Au cours des 12 derniers mois,36,2% des enquêtés avaient décidé de ne pas avoir de relations sexuelles et 27,8% s'isolaient de la famille et/ou de leurs amis. Plusieurs auteurs ont rapporté l'auto-stigmatisation chez les PvVIH [21, 25]. Le refus d'avoir des rapports sexuels par les PvVIH peut-être la conséquence de la stigmatisation dont ils sont victimes dans leur communauté. En effet, la divulgation de statut dont sont victimes les PvVIH de la part des personnels soignants et de la part de membres de la communauté contribue à les exclure de la société avec comme conséquences un repli sur soi et l’éviction de toute relation y compris des relations sexuelles.

Aussi, dans notre étude,23,9% des 276 enquêtés ayant interrompu leur traitement au cours des 12 derniers mois l'avaient fait par crainte que quelqu'un découvre leur statut. Cette crainte traduit une peur de la stigmatisation sociale dont peuvent être victimes les PvVIH puisque les centres de santé publics ou privés dispensant les ARV sont également fréquentés par la population générale qui peut découvrir la séropositivité des PvVIH.

Seuls 3,9% s’étaient vu refuser des soins de santé à cause de leur séropositivité, et 2,6% avaient évoqué l’évitement de tout contact physique ou la prise de mesures de précaution supplémentaires par les personnels de santé lors de l'administration des soins (comme porter des gants en double). Nos résultats sont semblables à ceux rapportés en Côte d'Ivoire [15] où 2,4% de PvVIH se sont vu refuser l'accès aux services de santé à cause de leur statut sérologique. Ces comportements de stigmatisation et de discrimination des prestataires de soins envers les PvVIH peuvent avoir comme conséquence une altération de leur état de santé tant physique que mentale. Plusieurs auteurs ont évoqué la crainte de la stigmatisation et de la discrimination comme pouvant freiner la recherche des soins de santé, limiter des efforts de prévention et même favoriser des troubles mentaux [5, 10, 16, 17, 19].

La plupart des comportements de stigmatisation – refus de service de santé (p = 0,04), violences verbales (p = 0,001) ou physiques (p = 0,01) – étaient significativement associés au sexe féminin. D'une façon générale, les femmes sont plus victimes de stigmatisations/discriminations [3]. Les violences basées sur le genre constituent donc une double peine pour les femmes infectées par le VIH.

En matière de santé sexuelle,2,1% des enquêtés avaient été conseillés de ne pas devenir mère/père d'un enfant et 1,4% avaient vu leur accès aux traitements ARV conditionné par l'utilisation d'une méthode contraceptive. Nos résultats sont inférieurs à ceux observés en Côte d'Ivoire en 2016 [15], où 3,1% des PvVIH affirment que leur capacité à obtenir des ARV était conditionnée par l'utilisation de certaines formes de contraception. Le conditionnement de l'accès aux ARV par l'utilisation de contraception peut conduire les PvVIH à ne plus prendre leur traitement, avec des conséquences négatives sur leur état de santé et des risques élevés de propagation du VIH. Aussi, le refus à une personne de devenir père/mère à cause de la séropositivité constitue une atteinte à la dignité de la personne, notamment dans les sociétés africaines. Dans l’étude de Fauk *et al.* [4], les PvVIH avaient comme attitude de ne pas révéler leur statut sérologique au personnel de santé lorsqu'elles se rendaient dans une structure de soins, de ne pas accéder à des services de santé dans des établissements autres que celui de leur suivi, ou de consulter uniquement des personnels de santé dont elles attendent un bon traitement [4]. En 2016 en Côte d'Ivoire [15],5,2% des PvVIH se sont vu refuser l'accès aux services de santé sexuelle et reproductive.

## Conclusion

La stigmatisation et la discrimination des PvVIH de la part des personnels soignants restent d'actualité au Togo, bien qu’à une fréquence relativement faible. Ces stigmatisations/discriminations peuvent avoir des conséquences néfastes sur la prise en charge globale des PvVIH et surtout sur l'accessibilité aux soins et la qualité des services délivrés.

Elles sont également responsables de comportements d'auto-stigmatisation des PvVIH aboutissant à une réduction de la fréquentation des structures de soins. Il est donc important de repenser les actions de prévention et d'intégrer un volet psychologique dans la prise en charge des PvVIH au Togo, mais aussi de mettre en place des centres de prise en charge spécialisés associés à la formation continue des personnels de santé afin d'atteindre les objectifs mondiaux en matière de lutte contre le VIH/sida.

## Financement

Cette étude a été financée par l'ONUSIDA, Global Network of People Living with HIV (GNP+) et l'USAID dans le cadre de la mise en œuvre du Plan stratégique national de lutte contre le sida au Togo.

## Contribution Des Auteurs

TECLESSSOU JN, AKAKPO AS: rédaction du manuscrit et finalisation.

DOKLA AK, AMOUSSOU KD, DEKU K, LIMAZIE CA: coordination scientifique générale de l’étude, analyse et l'interprétation des données et préparation du manuscrit final.

PITCHE P: responsable de la coordination scientifique globale de l’étude, rédaction du manuscrit et finalisation.

Tous les auteurs ont lu et approuvé le manuscrit.

## Liens D'intérêt

Les auteurs ne déclarent aucun lien d'intérêt
